# Copy Number Profiling of Brazilian Astrocytomas

**DOI:** 10.1534/g3.116.029884

**Published:** 2016-04-25

**Authors:** Lucas Tadeu Bidinotto, Raul Torrieri, Alan Mackay, Gisele Caravina Almeida, Marta Viana-Pereira, Adriana Cruvinel-Carloni, Maria Luisa Spina, Nathalia Cristina Campanella, Weder Pereira de Menezes, Carlos Afonso Clara, Aline Paixão Becker, Chris Jones, Rui Manuel Reis

**Affiliations:** * Molecular Oncology Research Center, Barretos Cancer Hospital, São Paulo, 14784 400, Brazil; † Barretos School of Health Sciences, Dr. Paulo Prata – FACISB, São Paulo, 14785 002, Brazil; ‡ Division of Molecular Pathology, The Institute of Cancer Research, London, SM2 5NG, UK; § Division of Cancer Therapeutics, The Institute of Cancer Research, London, SM2 5NG, UK; ** Department of Pathology, Barretos Cancer Hospital, São Paulo, 14784 400, Brazil; †† Life and Health Sciences Research Institute (ICVS), School of Health Sciences, University of Minho, Braga, 4704 553, Portugal; ‡‡ 3B’s – PT Government Associate Laboratory, Braga/Guimarães, 4704 553, Portugal; §§ Department of Neurosurgery, Barretos Cancer Hospital, São Paulo, 14784 400, Brazil

**Keywords:** genomics, glioblastomas, gliomas, *TERT*, *IDH1*

## Abstract

Copy number alterations (CNA) are one of the driving mechanisms of glioma tumorigenesis, and are currently used as important biomarkers in the routine setting. Therefore, we performed CNA profiling of 65 astrocytomas of distinct malignant grades (WHO grade I–IV) of Brazilian origin, using array-CGH and microsatellite instability analysis (MSI), and investigated their correlation with *TERT* and *IDH1* mutational status and clinico-pathological features. Furthermore, *in silico* analysis using the Oncomine database was performed to validate our findings and extend the findings to gene expression level. We found that the number of genomic alterations increases in accordance with glioma grade. In glioblastomas (GBM), the most common alterations were gene amplifications (*PDGFRA*, *KIT*, *KDR*, *EGFR*, and *MET)* and deletions (*CDKN2A* and *PTEN)*. Log-rank analysis correlated *EGFR* amplification and/or chr7 gain with better survival of the patients. MSI was observed in 11% of GBMs. A total of 69% of GBMs presented *TERT* mutation, whereas *IDH1* mutation was most frequent in diffuse (85.7%) and anaplastic (100%) astrocytomas. The combination of 1p19q deletion and *TERT* and *IDH1* mutational status separated tumor groups that showed distinct age of diagnosis and outcome. *In silico* validation pointed to less explored genes that may be worthy of future investigation, such as *CDK2*, *DMRTA1*, and *MTAP*. Herein, using an extensive integrated analysis, we indicated potentially important genes, not extensively studied in gliomas, that could be further explored to assess their biological and clinical impact in astrocytomas.

Malignant gliomas are highly invasive tumors that account for ∼70% of all primary adult brain neoplasms (Louis *et al.* 2007). Gliomas are classified into different histological subtypes, with astrocytomas the most prevalent. The WHO divides astrocytomas into four malignancy grades: pilocytic astrocytoma (WHO grade I), diffuse astrocytoma (WHO grade II), anaplastic astrocytoma (WHO grade III), and GBM (WHO grade IV) (Louis *et al.* 2007), the latter responsible for 82% of the malignant gliomas. GBMs are lethal tumors, presenting a one-year survival of 35.7%, and a five-year survival of 4.7% (Omuro and DeAngelis 2013). Clinically, GBM can be subdivided as: primary GBMs (approximately 95% of cases) when they arise *de novo* and typically manifest in older patients (peak age at diagnosis between 75 and 84 yr); or secondary GBMs, responsible for approximately 5% of all GBMs, which occur in younger patients, and can evolve from a lower-grade diffuse and/or an anaplastic astrocytoma (Sturm *et al.* 2014).

These clinical differences also reflect distinct genetic pathways; primary GBMs are characterized by *EGFR* amplification, and loss of heterozygosity (LOH) of chr10q, deletion of *PTEN*, and *p16*, whereas secondary GBMs are characterized by mutations in *TP53*, overexpression of *PDGFR*, LOH of chr10q, and abnormalities in the *p16* and *RB* pathways (Wen and Kesari 2008). Using an integrated genomics approach, the TCGA (The Cancer Genome Atlas) consortium described four different subtypes of GBMs (classical, mesenchymal, proneural, and neural) (Verhaak *et al.* 2010). Alterations (expression, mutation, and/or copy number) of the genes *TP53*, *IDH1*, *PDGFRA*, *EGFR*, *NF1*, and *CDKN2A* were considered the most important events to distinguish these four subtypes. Additional analysis of glioma–CpG island methylator phenotype (G–CIMP) positive and G–CIMP negative tumors has shown that DNA methylation patterns strongly correspond to the status of *IDH1* mutation (Noushmehr *et al.* 2010). Recently, hotspot *TERT* promoter gene mutations have been found in gliomas, with the highest incidence in GBMs (∼60%) (Vinagre *et al.* 2013; Heidenreich *et al.* 2014; Killela *et al.* 2013; Batista *et al.* 2016). These mutations generate a *de novo* binding site for GABPA transcription factor, which ultimately leads to high *TERT* expression (Bell *et al.* 2015). More recently, a large cohort study described five glioma groups based on 1p/19q codeletion, *IDH1/2* and *TERT* promoter mutational profile, with important clinical impact, with the “triple-negative” group or the only *TERT*-mutated group exhibiting a higher mortality risk (Foote *et al.* 2015; Eckel-Passow *et al.* 2015).

Therefore, the aim of this study was to characterize the genomic profile of 65 Brazilian astrocytomas, using aCGH and MSI, as well as to associate these data with the mutational status of the *TERT* promoter and *IDH1* genes, and clinico-pathological features of the patients. Additionally, by extending these analyses using *in silico* approaches, this study aimed to describe potentially important molecular subgroups with clinical impact and targets that could be the object of future investigation.

## Materials and Methods

### Patients

Sixty-five frozen tissue specimens comprising pilocytic astrocytomas (*n* = 7), diffuse astrocytomas (*n* = 9), anaplastic astrocytomas (*n* = 7), and GBMs (*n* = 41 primary and one secondary GBMs) were evaluated. Overall, there were 62 primary tumors and three recurrences (with the matched primary tumor also present in our analysis): one pilocytic astrocytoma that recurred after the first surgery, one diffuse astrocytoma that progressed to GBM after the surgery, and one GBM that recurred.

Histologic review of the slides was performed by two neuropathologists (A.P.B. and G.C.A.) to confirm the diagnosis, and to select the samples with > 75% of neoplastic cells and an absence of necrosis. DNA was isolated from frozen tissue and the peripheral blood of each patient and used for further analysis. Clinical data for each patient was obtained, and the summary of the characteristics is shown in Table 1. The present study was approved by the Barretos Cancer Hospital Ethical Committee (ID 408/2010).

**Table 1:** Clinico-pathological features of astrocytomas

		Pilocytic Astrocytoma	Diffuse Astrocytoma	Anaplastic Astrocytoma	Glioblastoma
Number of patients		7	9	7	42
Age (years)*^a^*		16.7 (9–38)	38.5 (15–70)	35.7 (30–44)	59.4 (25–81)
Sex	Male	85.7%	44.4%	42.9%	66.7%
	Female	14.3%	55.6%	57.1%	33.3%
Follow up (months)*^a^*		39.7 (17–56)	27 (0–58)	36.6 (0–93)	8.5 (0–43)
Karnofsky Performance Status (KPS)	< 70	0%	0%	14.3%	28.6%
	≥ 70	100%	100%	85.7%	59.5%
	N/A	0%	0%	0%	11.9%
Surgery type	Total resection	42.9%	22.2%	57.1%	47.6%
	Partial resection	42.9%	44.4%	14.3%	47.6%
	N/A	14.2%	33.4%	28.6%	4.8%
Radiotherapy	Yes	0%	22.2%	71.4%	54.8%
	No	100%	77.8%	28.6%	45.2%
Chemotherapy	Yes	0%	0%	14.3%	26.2%
	No	100%	100%	85.7%	73.8%
Status of the patient	Alive, free of disease	14.3%	11.1%	0%	0%
	Alive, with the disease	85.7%	44.5%	57.1%	16.7%
	Death by cancer	0%	33.3%	42.9%	81%
	N/A	0%	11.1%	0%	2.3%

N/A, not available.

a Average (minimum–maximum).

### DNA isolation

The DNA from patients’ blood was isolated using a QIAmp DNA blood Mini Kit (Qiagen), and DNA from frozen tumor tissue was isolated using a DNeasy Blood and Tissue Kit (Qiagen) according to the protocols provided by the supplier. The 260/280 and 260/230 ratios were determined by NanoDrop (Thermo Scientific) and the DNA was quantified using Quant-IT PicoGreen dsDNA (Invitrogen), using the supplier’s protocol.

### Array-CGH

Two-color 60 K array Comparative Genomic Hybridization (aCGH) was performed using the default protocol published by Agilent Technologies (Agilent Oligonucleotide Array-Based CGH for Genomic DNA Analysis Enzymatic Labeling for Blood, Cells, or Tissues, protocol v. 7.2, published in July 2012) as previously described (Bidinotto *et al.* 2015). DNA of each patient’s blood was used as control, in order to exclude copy number variations. *AluI* and *RsaI* restriction enzymes were used to digest 400 ng of both tumor and blood DNA, which was then incubated with random primers. Blood DNA was labeled with cyanine-3 (Cy3), whereas tumor DNA was labeled with cyanine-5 (Cy5). Equal quantities of Cy3- and Cy5-labeled DNA was hybridized into Agilent Human Genome CGH 8 × 60 K microarray slides overnight, and washed according to the supplier’s default protocol. The slides were scanned and decoded by the software Feature Extraction v. 10.7 (Agilent Technologies), using the protocol CGH_107_Sep09. The signal intensities were log2 transformed, and the spots were mapped in the most recent version of the human genome (hg19). The data were Lowess normalized and smoothing corrected. The data were CBS segmented. The low-level copy gains/losses threshold value was considered 0.1, and the moderate-to-high gene amplification/homozygous deletion threshold value was considered 0.7, in five consecutive probes. aCGH data of the 65 Agilent arrays can be accessed using the Gene Expression Omnibus (GEO) series accession number GSE71538 (http://www.ncbi.nlm.nih.gov/geo/query/acc.cgi?acc=GSE71538).

### Bioinformatics analysis

Genome plots of each case were generated and visually inspected. Next, frequency plots were generated, and genomic regions were considered as frequently altered when they were gained or lost in at least 30% of the same tumor type. Survival analysis was performed to each altered region found in GBM samples. Kaplan–Meier plots were done considering the regions statistically significant (*P* < 0.05) in log rank tests. In order to validate our findings, we extended our aCGH analysis using publicly available GBM data on the TCGA Research Network dataset (http://cancergenome.nih.gov). The set consisted of 498 GBMs CNV MSKCC level 1 data of the Agilent Human Genome CGH 244K microarray platform, and it was subjected to the same CNA detection algorithms performed in our samples.

Genomic regions frequently amplified or deleted were considered for further *in silico* analysis using the professional version of the compendium of cancer transcriptome profiles, Oncomine (Compendia Bioscience, Ann Arbor, MI). Eight GBM expression datasets (totalizing 1489 tumors and brain normal samples) were selected from the Oncomine database. The expression of potentially relevant genes was analyzed in these datasets by selecting the genes that were present in frequently amplified or deleted regions of our aCGH experiments. The genes considered relevant presented amplification in our experiment and overexpression in the Oncomine datasets, or homozygous deletion in our experiment and loss of expression in the Oncomine datasets.

The mRNA expression of these relevant genes was further assessed on the TCGA Research Network GBM expression dataset (http://cancergenome.nih.gov, *n* = 542 GBMs) from the Oncomine database. The expression of the genes was categorized in terms of positive or negative for each patient, based on a median intensity of log2 median-centered value. If the intensity value of the gene was greater than the median value considering the 542 GBMs, it was considered positive; otherwise, the gene was considered negative in the patient. Furthermore, the expression of each gene was correlated to the overall survival of the patients.

Additionally, correlation studies were performed. The genes were PCA ordered, and Pearson correlation coefficient was assessed on the TCGA dataset. Correlations with *P* < 0.05 were considered statistically significant.

Finally, the potentially relevant genes were clustered by biological importance and canonical pathways, using the DAVID v6.7 bioinformatics tool (The Database for Annotation, Visualization, and Integrated Discovery) (Huang *et al.* 2007).

### Microsatellite instability (MSI)

The MSI analysis of tumor and blood DNA of the patients was performed according to methodology previously published (Viana-Pereira *et al.* 2011; Campanella *et al.* 2014, 2015a). Briefly, five markers (NR27, NR21, NR24, BAT25, and BAT26) were PCR multiplexed, and the products were separated using an ABI Prism 3500 genetic analyzer (Life Technologies). The results were analyzed with GeneScan Analysis software, version 3.7 (Life Technologies).

### TERT mutation analysis

The hotspot mutations analysis of the *TERT* promoter gene was performed by PCR followed by direct Sanger sequencing (Vinagre *et al.* 2013; Campanella *et al.* 2015b; Batista *et al.* 2016). Briefly, the *TERT* promoter region was amplified by PCR using the primers: 5′-AGTGGATTCGCGGGCACAGA-3′ (forward) and 5′-CAGCGCTGCCTGAAACTC-3′ (reverse), leading to a 235 bp PCR product containing the C228T and C250T mutations. Amplification PCR was performed with an initial denaturation at 95° for 15 min, followed by 40 cycles of 95° denaturation for 30 sec, 64° annealing for 90 sec, and 72° elongation for 30 sec, and 72° final elongation for 7 min. Amplification of PCR products was confirmed by gel electrophoresis. Sequencing PCR was performed using a Big Dye terminator v3.1 cycle sequencing ready reaction kit (Applied Biosystems) and an ABI PRISM 3500 xL Genetic Analyzer (Applied Biosystems).

### IDH1 mutation analysis

The analysis of hotspot mutations of *IDH1* (exon 4) was performed by PCR followed by direct sequencing. Briefly, the *IDH1* region of interest was amplified by PCR using the primers: 5′-CGGTCTTCAGAGAAGCCATT-3′ (forward) and 5′-CACATTATTGCCAACATGAC-3′ (reverse). An amplification PCR reaction was performed in a total volume of 15 μl, comprising: 1 μl of DNA, 1 × buffer solution, 2 mM MgCl_2_, 200 μM of each dNTP, 0.3 μM of each set primer, and 0.5 U Taq DNA polymerase (Invitrogen), and was performed in a Veriti 96-well Thermal Cycler with an initial denaturation at 95° for 10 min, amplified for 40 cycles of denaturation at 95° for 45 sec, annealing at 58° for 45 sec, and extension at 72° for 45 sec, and a final extension at 72° for 10 min. Amplification of PCR products was confirmed by gel electrophoresis. Sequencing PCR was performed using a Big Dye terminator v3.1 cycle sequencing ready reaction kit (Applied Biosystems) and an ABI PRISM 3500 xL Genetic Analyzer (Applied Biosystems).

### Data availability

The authors state that all data necessary for confirming the conclusions presented in the article are represented fully within the article.

## Results

### Copy number alterations (CNA)

**Figure 1:**
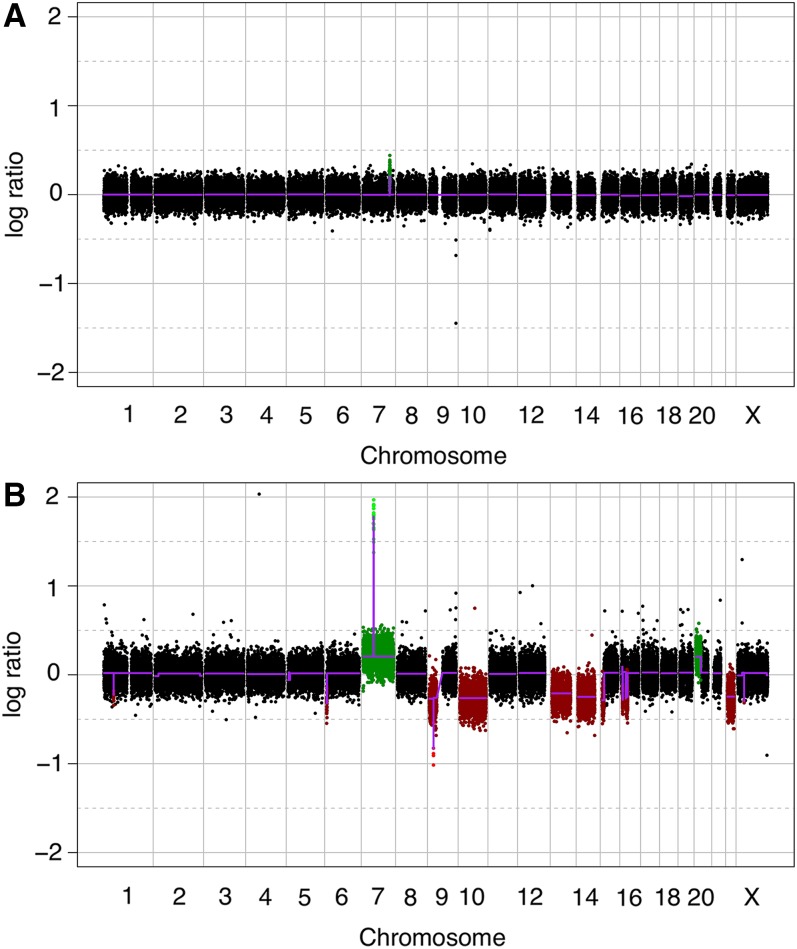
Plots representing the whole genome of (A) pilocytic astrocytoma and (B) glioblastoma.

The number of genomic alterations, detected through aCGH, increased in accordance with WHO grade. The average number of CNAs per sample varied from 5.4 in pilocytic astrocytomas (Figure 1A) to 33.8 in GBMs (Figure 1B). The average number of gains, losses, amplifications, and deletions per tumor type is described in Table 2.

**Table 2:** Average number of alterations in aCGH cases

Tumor Type	Gains	Losses	Amplifications	Deletions	Total Number of Alterations
Pilocytic astrocytoma	2.6 (0–9)	2.7 (0–11)	0	0.1 (0–1)	5.4 (0–20)
Diffuse astrocytoma	4.7 (0–11)	5.8 (2–12)	0.2 (0–1)	0.1 (0–1)	10.8 (4–22)
Anaplastic astrocytoma	8.0 (1–12)	10.1 (3–21)	0	0	18.1 (11–33)
Glioblastoma	14.0 (0–90)	17.4 (2–61)	1.5 (0–10)	0.9 (0–4)	33.8 (7–164)

Values expressed as average (minimum–maximum) in each case. aCGH, array comparative genomic hybridization.

**Figure 2:**
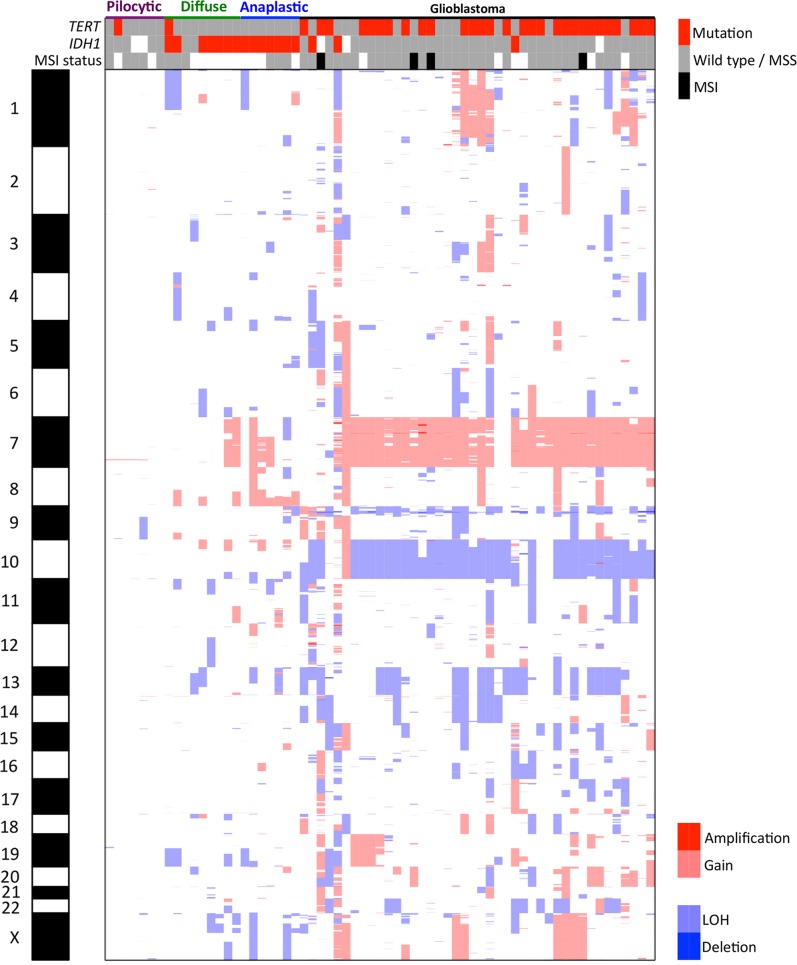
Heatmap representing the amplifications, gains, losses, and deletions detected through aCGH in pilocytic astrocytomas, diffuse astrocytomas, anaplastic astrocytomas and glioblastomas. aCGH, array comparative genomic hybridization; LOH, loss of heterozygosity; MSI, microsatellite instability analysis; MSS, microsatellite stable.

**Figure 3:**
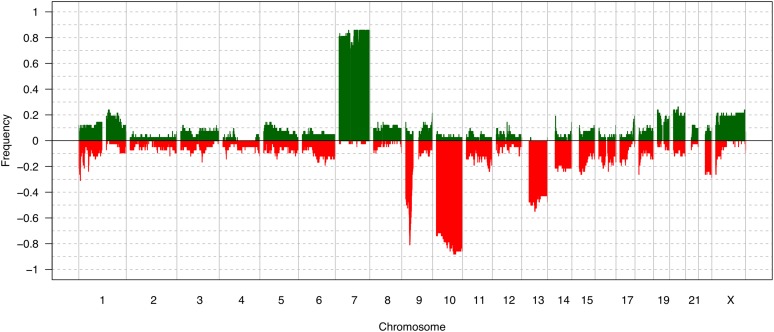
Frequency plot representing the gained and lost regions in glioblastomas.

The summary of gains, losses, amplifications, and deletions in all the samples is found in Figure 2. In pilocytic astrocytomas (WHO grade I), we detected gain of chr7q34, which was associated with the presence of the *KIAA1549:BRAF* gene fusion, as recently reported by our group via FISH assay on these samples (Becker *et al.* 2015a). In diffuse astrocytomas (WHO grade II), the most frequent alterations were gains in chr7q31.1, chr8q23.3-q24.3, chr14q11.2, and chr17q25.3, and losses in chr1p36.33-p36.32, 1p35.3, chr19q13.31-q13.43, chrXp22.33-p21.3, and chrXp11.4-p11.22. In anaplastic astrocytomas (WHO grade III), the most frequent alterations were gains in chr7q11.23-q35, chr8q21.3-q24.3, and chr10p15.3-p14, and losses in chr1p36.33-p36.32, chr2q37.1-q37.3, chr4q35.1-q35.2, chr11p15.5-p15.4, chr13q11-q34, chrXp22.33-p22.31, and chrXp11.4-p11.2. The overall alterations identified in GBMs are summarized in Figure 3. The most recurrent alterations were gain in chr7p22.3-q36.3, and losses in chr9p24.3-p21.1 and chr10p15.3-q26.3. Frequent losses were also observed at chr13q11.q34, chr14q11.2, and chr22q11.1-q13. Amplification of the chr7p12.2-p11.2 region was found in 33.3% of the GBMs, and 47.6% of cases exhibited deletion of chr9p22.1-p21.3. Loss of the 10q23.2-q23.31 region was found in 88.1% of cases. However, only a small fraction (4.8%) presented homozygous deletion, and 31% of the samples presented loss in 1p32.3 (Figure 3). Due to the high prevalence of alterations, we further evaluated the candidate genes present in these regions (Table 3).

**Table 3:** Most frequently amplified and deleted regions in glioblastoma samples

Event	*N*	Region	Genes
Amplification	2	4q11-q12	*DCUN1D4*, *LRRC66*, *SGCB*, *SPATA18*, *SCFD2*, *FIP1L1*, *LNX1*, *CHIC2*, *GSX2*, ***PDGFRA***, ***KIT***, ***KDR***, *SRD5A3*, *TMEM165*, *CLOCK*, *PDCL2*
Amplification	14	7p12.2-p11.2	*VWC2*, *ZPBP*, *IKZF1*, *FIGNL1*, *DDC*, *GRB10*, *COBL*, *POM121L12*, *VSTM2A*, *SEC61G*, ***EGFR***, *LANCL2*, *SEPT14*, *ZNF713*, *GBAS*, *PSPH*, *CCT6A*, *SUMF2*, *PHKG1*, *CHCHD2*
Amplification	2	7q31.2	*CAV2*, *CAV1*, *MET*, *CAPZA2*
Amplification	4	12q13.2-q13.3	*NEUROD4*, *OR9K2*, *OR10A7*, *OR6C74*, *OR6C6*, *OR6C1*, *OR6C3*, *OR6C75*, *OR6C65*, *PHC1B*, *OR6C76*, *OR6C2*, *OR6C70*, *OR6C68*, *OR6C4*, *OR2AP1*, *OR10P1*, *METTL7B*, *ITGA7*, *BLOC1S1*, *RDH5*, *CD63*, *GDF11*, *SARNP*, *ORMDL2*, *DNAJC14*, *MMP19*, *WIBG*, *DGKA*, *SILV*, ***CDK2***, *RAB5B*, *SUOX*, *IKZF4*, *RPS26*, *ERBB3*, *PA2G4*, *ZC3H10*, *FAM62A*, *MYL6*, *SMARCC2*, *RNF41*, *OBFC2B*, *SLC39A5*, *ANKRD52*, *COQ10A*, *CS*, *CNPY2*, *PAN2*, *IL23A*, *STAT2*, *APOF*, *TIMELESS*, *MIP*, *SPRYD4*, *GLS2*, *RBMS2*, *BAZ2A*, *ATP5B*, *PTGES3*, *NACA*, *PRIM1*
Amplification	3	12q14.3-q15	*CAND1*, *DYRK2*
Deletion	2	1p32.3	***DMRTA2***, *FAF1*
Deletion	20	9p22.1-p21.3	*SLC24A2*, *MLLT3*, *KIAA1797*, *PTPLAD2*, ***IFNB1***, ***IFNW1***, ***IFNA21***, ***IFNA4***, ***IFNA7***, ***IFNA10***, ***IFNA16***, ***IFNA17***, ***IFNA14***, ***IFNA5***, *KLHL9*, ***IFNA6***, ***IFNA13***, ***IFNA2***, ***IFNA8***, ***IFNA1***, ***MTAP***, *C9orf53*, ***CDKN2A***, ***CDKN2B***, ***DMRTA1***, *ELAVL2*, *C9orf134*
Deletion	2	10q23.2-q23.31	*PAPSS2*, *ATAD1*, ***PTEN***

The genes of potential importance are shown in bold. *N*, number of GBM cases.

### TERT and IDH1 mutation

From the 65 samples analyzed for *TERT* mutation, 47.7% were mutated (33.9% at the c.-124C > T hotspot, and 13.8% at c.-146C > T) (Figure 2) and 52.3% were wild-type. The percentage of mutated samples was 14.3% (1/7) in pilocytic astrocytomas, 11.1% (1/9) in diffuse astrocytomas, 0% (0/7) in anaplastic astrocytomas, and 69.1% (29/42) in GBMs (Supplemental Material, Table S1).

*IDH1* mutational status was assessed in 60 samples (five pilocytic astrocytomas, nine diffuse astrocytomas, seven anaplastic astrocytomas, and 39 GBMs), and we observed that 17/60 (28.3%) of cases presented *IDH1* mutation. The percentage of mutated samples in the tumor types was 0% in pilocytic astrocytomas, 77.8% in diffuse astrocytomas (six presenting Arg132His and one presenting Arg132Cis mutation), 100% in anaplastic astrocytomas (five presenting Arg132His and two Arg132Cis mutation), and 7.7% in GBMs (all presenting Arg132His mutation) (Figure 2 and Table S1).

### Microsatellite analysis

MSI status was assessed in 55 cases (Table 4); MSI-H was only observed in four GBMs (11.1%), the remaining samples presenting microsatellite stable or MSI-L phenotypes (Table 4). One MSI case presented a high number of CNAs (total of 81 CNAs) and *TERT* mutation. Two other samples presented *TERT* mutation, were wild-type for *IDH1*, and presented a total of 23 and 24 CNAs, respectively. The remaining sample was wild-type for *TERT* and *IDH1*, and presented 25 CNAs.

**Table 4:** Microsatellite stability status of the glioma samples

Tumor Type	*N*	MSI Status	
		MSS + MSI-L	MSI-H
Pilocytic astrocytoma	6	6 (100%)	0
Diffuse astrocytoma	8	8 (100%)	0
Anaplastic astrocytoma	5	5 (100%)	0
Glioblastoma	36	32 (88.9%)	4 (11.1%)

*N*, number of samples analyzed of each tumor type; MSI, microsatellite instability; MSS, microsatellite stable; MSI-L, low microsatellite instability; MSI-H, high microsatellite instability.

### Matched primary recurrence tumors

**Figure 4:**
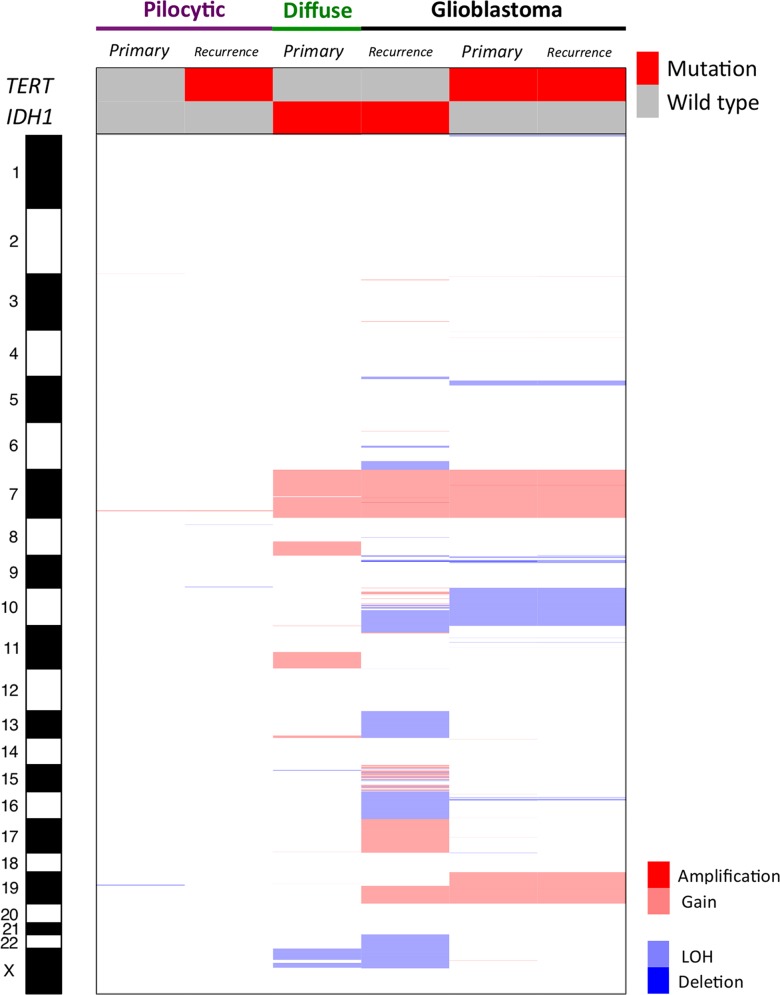
Heatmap representing the amplifications, gains, losses, and deletions through aCGH, as well as the mutational profile of *TERT* and *IDH1*, of the primary and matched recurrence tumors. aCGH, array comparative genomic hybridization; LOH, loss of heterozygosity.

The molecular profile of the matched primary and recurrence tumors are summarized in Figure 4.

The recurrence of pilocytic astrocytoma presented mutation in *TERT*. No other molecular differences were found when the primary pilocytic astrocytoma was compared to the recurrence (both presented only chr7q34 gain, with no other gene mutation found).

The primary diffuse astrocytoma presented mainly chr7, chr8q, and chr11q gains, as well as chrXp loss. At recurrence, it progressed to GBM, with the typical features of this tumor type described above (amplification of *EGFR*, gain at chr7, and losses at chr9p24.3-p21.1, chr10p15.3-q26.3, chr13, and chr22). ChrXp loss and *IDH1* mutation were found in both tumors (primary and recurrence).

Finally, the GBM sample that recurred into GBM presented exactly the same molecular features, being the typical chromosomal characteristics presented above, and *TERT* mutated. All these samples were microsatellite stable.

### Clinical impact of the molecular features

**Figure 5:**
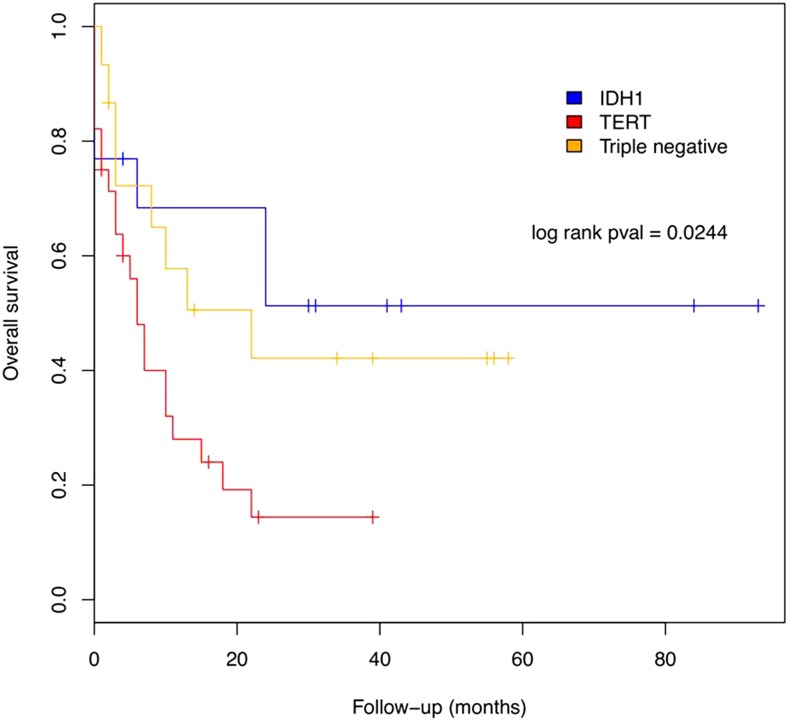
Survival curve considering the patients presenting only *IDH1* mutation, only *TERT* mutation, and neither mutation in *IDH1* nor *TERT*, nor loss of 1p19q (triple negative).

Following the criteria recently described (Eckel-Passow *et al.* 2015), we separated the cases based on 1p/19q deletion and *IDH1* and *TERT* promoter mutational status. We found that 1.8% of the cases presented the three alterations “triple positive,” 22.8% presented mutation only in *IDH1*, 49.1% presented mutation only in *TERT*, and 26.3% did not present alteration in any of these markers and was considered to be “triple negative” (Table 5). Of note, survival curves show that the group of cases with mutation only in *TERT* presented lower survival than those presenting only *IDH1* mutation (mean survival of 8.5 months *vs.* 29.2 months, respectively, *P* = 0.024 in log rank test), whereas “triple negative” cases presented a mean survival of 21.3 months (Figure 5).

**Table 5:** Percentage of cases, age at diagnosis and mean survival of the patients divided in molecular groups based on 1p19q deletion, *TERT* promoter and *IDH1* mutational status

Molecular Feature	Percentage of Cases	Age at Diagnosis (Years)	Mean Survival (Months)
Triple positive	1.8	42	55
*IDH1* only	22.8	38.8	29.2
*TERT* only	49.1	59.9	8.5
Triple negative	26.3	43.5	21.3

Triple positive represents 1p19q deletion + mutation in *TERT* promoter and *IDH1*; Triple negative represents none of the three alterations.

**Figure 6:**
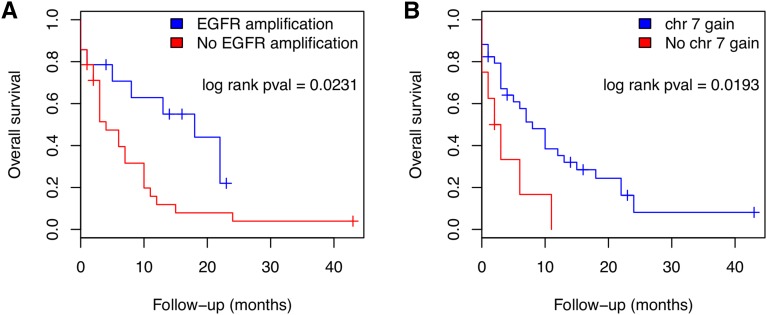
Survival curves of the patients considering (A) *EGFR* amplification and (B) chromosome 7 gain.

Nonsupervised hierarchical cluster analysis of the GBM CNA did not show any association with clinico-pathological features (data not shown). Log rank analysis of all altered regions across the GBM samples pointed to the correlation of *EGFR* amplification and/or gain of chr7 to better survival of patients (*P* < 0.05, Figure 6).

### In silico analysis

**Figure 7:**
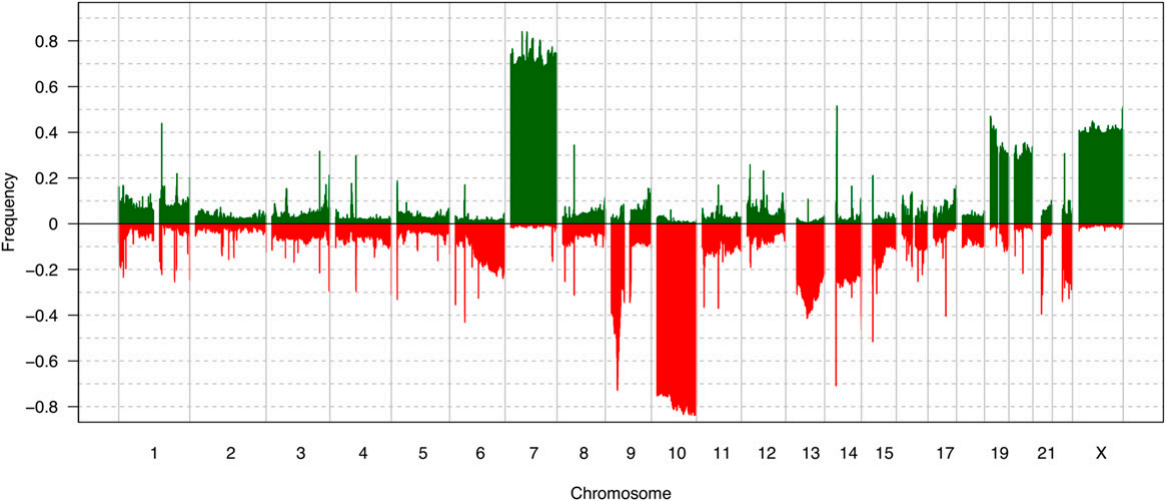
Frequency plot representing the gained and lost regions in The Cancer Genome Atlas (TCGA) glioblastoma dataset.

In order to validate our aCGH findings, TCGA analysis of 498 aCGH samples extended our GBM profiling of the Brazilian population (Figure 7). The most recurrent alterations were gain of chr7, chr19, chr20, and chrX, as well as losses of chr9p, chr10, and chr13. Amplification of chr7p14.1-q11.21 was found in 226 cases (45.4%) and deletion in 9p22.1-p21.1 was found in 176 cases (59%). A small fraction of the cases were found with deletion in chr10q23.2-q23.31 (25 cases - 5%), while 417 cases (83.7%) presented a loss in this region.

Moreover, we extended our findings to the expression level. The genes frequently found amplified/deleted in our GBM cases were investigated through a bioinformatics approach, using the compendium of cancer transcriptome profiles (Oncomine). Once the list of genes encompassed in the amplified or deleted regions of our GBM samples was generated, we inquired whether these genes had gain or loss of expression in eight other GBM expression datasets (totaling 1489 tumor and normal brain samples).

More than half of the amplified genes, with concomitant overexpression, were present in chr7p21.1-p11.2 (*TWIST1,* *FERD3L*, *TWISTNB*, *TMEM196*, *MACC1*, *ITGB8*, *SP8*, *SP4*, *DNAH11*, *CDCA7L*, *RAPGEF5*, *IL6*, *FAM126A*, *KLHL7*, *NUPL2*, *GPNMB*, *C7orf30*, *IGF2BP3*, *TRA2A*, *CCDC126*, *STK31*, *VWC2*, *FIGNL1*, *GRB10*, *SEC61G*, *EGFR*, *LANCL2*, *SEPT14*, *ZNF713*, *GBAS*, *PSPH*, *CCT6A*, *SUMF2*, *PHKG1*, and *CHCHD2*), whereas 90.9% of the deleted genes, presenting loss of expression were located at chr9p22.1-p21.3 (*SLC24A2*, *MLLT3*, *KIAA1797*, *PTPLAD2*, *IFNB1*, *IFNW1*, *IFNA21*, *IFNA4*, *IFNA7*, *IFNA17*, *IFNA14*, *IFNA5*, *IFNA6*, *IFNA13*, *IFNA8*, *IFNA1*, *MTAP*, *CDKN2A*, *DMRTA1*, and *ELAVL2*) (Table 6), suggesting the potential importance of the genes present in these regions in GBM development.

**Table 6:** Amplified genes that presented overexpression and deleted genes that presented decreased expression in Oncomine datasets

Event	Region	Genes
Amp/Overexp	4q11-q12	*LRRC66*, *SGCB*, *SPATA18*, *SCFD2*, *CHIC2*, *PDGFRA*, *KDR*
Amp/Overexp	7p21.1-p15.3	*TWIST1*, *FERD3L*, *TWISTNB*, *TMEM196*, *MACC1*, *ITGB8*, *SP8*, *SP4*, *DNAH11*, *CDCA7L*, *RAPGEF5*, *IL6*, *FAM126A*, *KLHL7*, *NUPL2*, *GPNMB*, *C7orf30*, *IGF2BP3*, *TRA2A*, *CCDC126*, *STK31*
Amp/Overexp	7p12.2-p11.2	*VWC2*, *FIGNL1*, *GRB10*, *SEC61G*, *EGFR*, *LANCL2*, *SEPT14*, *ZNF713*, *GBAS*, *PSPH*, *CCT6A*, *SUMF2*, *PHKG1*, *CHCHD2*
Amp/Overexp	7q31.2	*CAV2*, *CAV1*, *CAPZA2*
Amp/Overexp	12q13.2-q13.3	*OR6C1*, *OR6C68*, *OR6C4*, *OR10P1*, *ITGA7*, *BLOC1S1*, *GDF11*, *SARNP*, *DNAJC14*, *WIBG*, *CDK2*, *PA2G4*, *FAM62A*, *MYL6*, *SMARCC2*, *ANKRD52*, *CS*
Amp/Overexp	12q14.3-q15	*CAND1*, *DYRK2*
Del/LOexp	9p22.1-p21.3	*SLC24A2*, *MLLT3*, *KIAA1797*, *PTPLAD2*, *IFNB1*, *IFNW1*, *IFNA21*, *IFNA4*, *IFNA7*, *IFNA17*, *IFNA14*, *IFNA5*, *IFNA6*, *IFNA13*, *IFNA8*, *IFNA1*, *MTAP*, *CDKN2A*, *DMRTA1*, *ELAVL2*
Del/LOexp	10q23.2-q23.31	*ATAD1*, *PTEN*

Amp, amplification in GBM samples; Overexp, overexpression in Oncomine samples; Del, deletion in GBM samples; LOexp, loss of expression in Oncomine samples.

**Figure 8:**
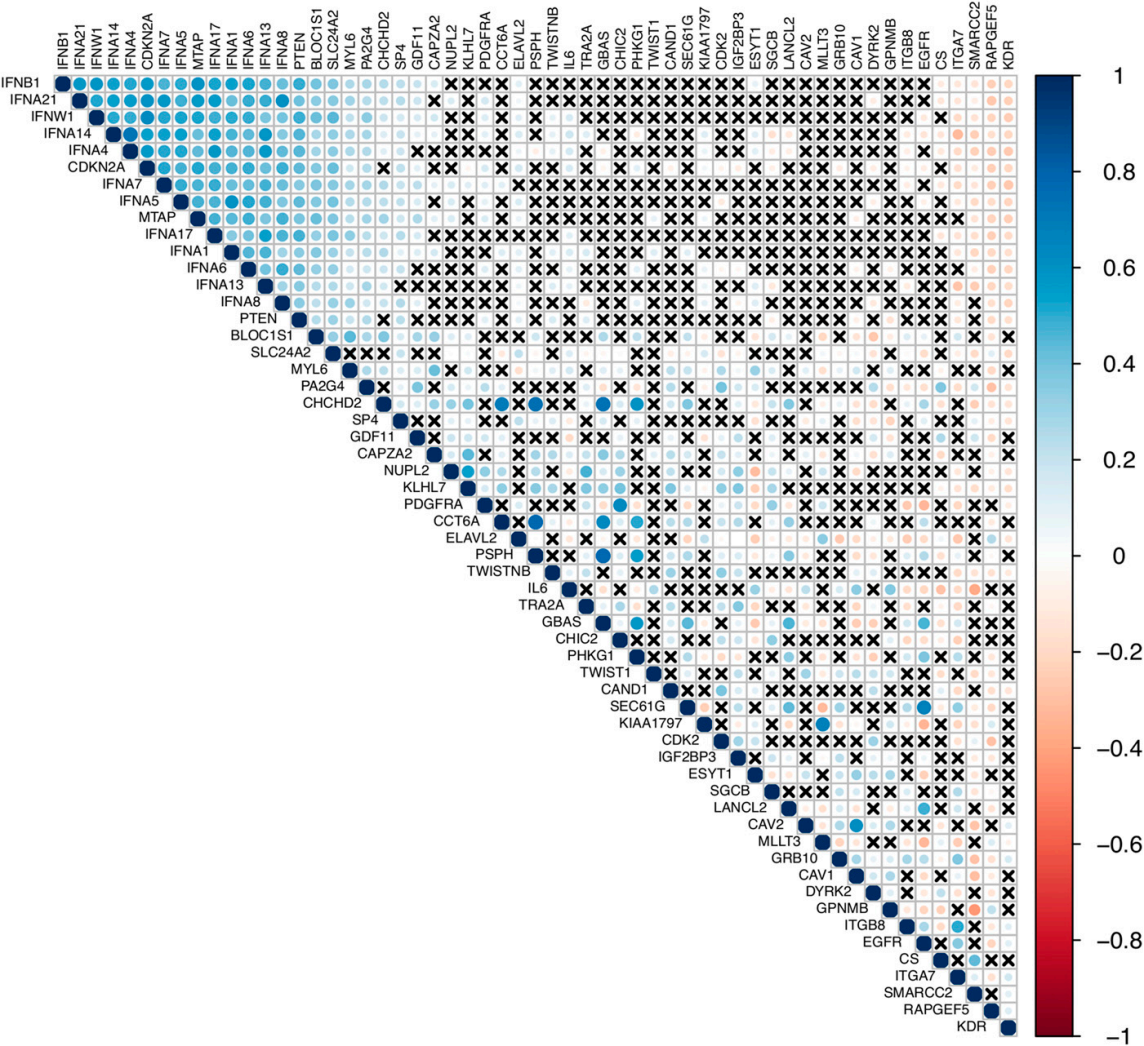
Correlation of the expression in TGCA The Cancer Genome Atlas dataset considering the genes deleted or amplified in our datasets. The crosses indicate that there was no statistical difference in the correlation.

Considering these potentially important genes (Table 6), we found in the TCGA expression dataset that the loss of expression of *IFNA13*, *IFNA21*, *IFNA6*, *IFNA8*, *IFNB1*, *IFNW1*, or *PTEN* was correlated with poor survival, whereas loss of expression of *GPNMB*, *IGF2BP3*, *ITGB8*, or *SEC61G* was correlated with better survival (Table 7). Additionally, we found that there is an important positive correlation of expression among the genes *IFNB1*, *IFNA21*, *IFNW1*, *IFNA14*, *IFNA4*, *CDKN2A*, *IFNA7*, *IFNA5*, *MTAP*, *IFNA17*, *IFNA1*, *IFNA6*, *IFNA13*, *IFNA8*, *PTEN*, *BLOC1S1*, *SLC24A2*, *MYL6*, *PA2G2*, *CHCHD2*, *SP4*, *GDF11*, and *CAPA2*. Furthermore, there is an important negative correlation of these genes with *CS*, *ITGA7*, *SMARCC2*, *RAPGEF5*, and *KDR* (Figure 8).

**Table 7:** Genes correlated to overall survival in the GBM samples

Gene	Genome Location	Log Rank *P* Value
*IFNA13**^a^*	9p22.1-p21.3	0.039
*IFNA21**^a^*	9p22.1-p21.3	0.01
*IFNA6**^a^*	9p22.1-p21.3	0.016
*IFNA8**^a^*	9p22.1-p21.3	0.002
*IFNB1**^a^*	9p22.1-p21.3	0.007
*IFNW1**^a^*	9p22.1-p21.3	0.002
*GPNMB**b*	7p21.1-p15.3	0.008
*IGF2BP3**b*	7p21.1-p15.3	0.008
*ITGB8**b*	7p21.1-p15.3	0.023
*SEC61G**b*	7p12.2-p11.2	0.000025
*PTEN**^a^*	10q23.2-q23.31	0.006

a Genes for which the loss of expression was correlated to poor survival.

b Genes for which the loss of expression was correlated to better survival.

**Figure 9:**
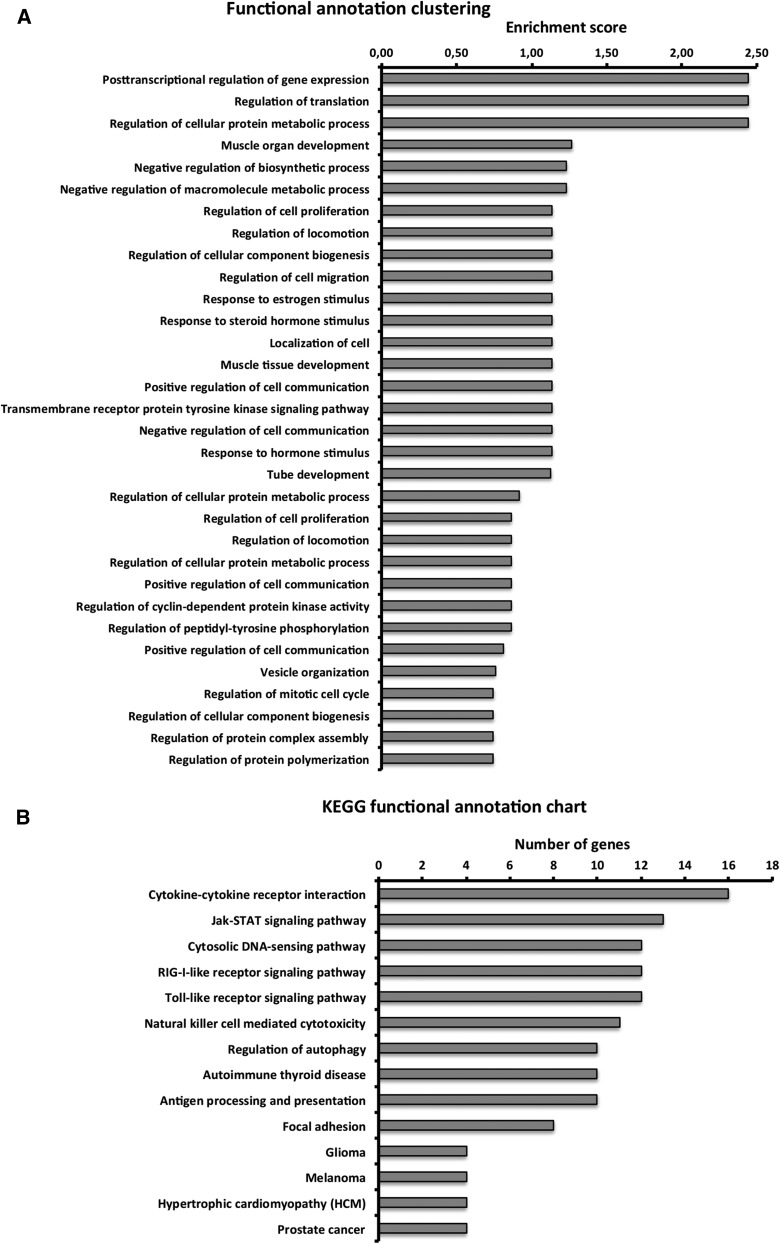
DAVID clustering analysis showing (A) functional annotation clustering based on biological processes and (B) Kyoto Encyclopedia of Genes and Genomes (KEGG) functional annotation. DAVID, The Database for Annotation, Visualization, and Integrated Discovery.

Finally, DAVID analysis showed that there are several functional annotation clusters with a high enrichment score related to potentially important biological processes, such as posttranscriptional regulation of gene expression, regulation of translation, regulation of cell proliferation, and the transmembrane receptor protein tyrosine kinase signaling pathway (Figure 9A). KEGG canonical pathways with a high number of genes include the Jak-STAT signaling pathway, alongside pathways related to the immune response, glioma, and prostate cancer (Figure 9B).

## Discussion

In the present study, we performed a molecular characterization in order to describe the genomic alterations and mutation status of the key *TERT* and *IDH1* genes in astrocytomas arising in the Brazilian population.

Overall, the CNAs found in our aCGH analysis correspond to the alterations found in the TCGA datasets. We found that 85.7% of the GBMs presented gain of whole chr7, and 33.3% presented amplification in the region 7p12.2-p11.2, in which *EGFR* is included. The *PTEN* tumor suppressor gene loci exhibited loss in 88.1% of our samples. From the 42 GBM samples, only one did not present alterations in chr7 and/or chr10, showing the importance of these loci in gliomagenesis. Log rank analysis showed that our patients presenting *EGFR* amplification and/or chr7 gain had better overall survival than patients that did not present these alterations. This finding is in accordance with Smith *et al.* (2001) and Verhaak *et al.* (2010), who subdivided the GBMs into four groups and found that patients with the ‘classical’ subtype, characterized by *EGFR* amplification and chr10 loss, presented better overall survival when they receive an intensive therapy (concurrent chemo- and radiotherapy or more than three subsequent cycles of chemotherapy).

By analyzing the TCGA expression dataset, we found several other genes correlated with overall survival. In order to determine the possible interference of the coexpression of the genes in these results, we performed correlation tests in potentially important genes in GBM development. More than half of the genes that were statistically significant were located on 9p22.1-p21.3. This locus a frequent target of homozygous deletion during gliomagenesis, and this event was observed in more than half of our patients. This region encompasses the *CDKN2A* tumor suppressor gene (*p16^INK4a^*/*p14^ARF^*/*p15^INK4b^* locus), a potent regulator of the cell cycle (Li *et al.* 2011). Of interest, the chr13q region encompassing the *RB1* gene presented a loss in 57.1% of our GBM samples. Previous studies have reported that homozygous deletion of *p16^INK4a^*, *CDK4* amplification, and loss of *RB1* are almost mutually exclusive (Verhaak *et al.* 2010; Ohgaki and Kleihues 2009), and that these alterations are found in ∼50% of primary GBMs (Ohgaki and Kleihues 2009).

We have previously determined MSI status in 144 gliomas (71 children and young people and 73 adults). Of the 14 gliomas that were from patients of Brazilian origin, all of them were < 18 years of age (Viana-Pereira *et al.* 2011). Overall, a total of 13.2% of the samples presented MSI, in which the majority was pediatric (*P* = 0.02, Chi-square test). Similar to the findings of the present work, all the adult MSI-positive cases previously reported were GBMs (Viana-Pereira *et al.* 2011).

Recurrent mutations in the promoter region of *TERT* gene, namely the c.-146:C > T and the c.-124:C > T mutations, were recently reported in several tumors, including melanomas, bladder, hepatocarcinoma, thyroid carcinomas, and gliomas (Huang *et al.* 2013; Vinagre *et al.* 2013; Killela *et al.* 2013; Horn *et al.* 2013; Bell *et al.* 2015; Batista *et al.* 2016). These mutations generate a consensus binding site for ETS/TCF transcription factors (CCGGAA), resulting in increased activity of the *TERT* promoter and abnormal telomere size maintenance (Huang *et al.* 2013; Vinagre *et al.* 2013; Killela *et al.* 2013; Horn *et al.* 2013; Bell *et al.* 2015; Cancer Genome Atlas Research Network *et al.* 2015; Eckel-Passow *et al.* 2015; Koelsche *et al.* 2013). In accordance, we found a low percentage of pilocytic, diffuse, and anaplastic astrocytomas presenting mutations in the *TERT* promoter gene. Additionally, we found a high percentage of GBMs presenting either the mutation -124:G > A (52.6%) or -146:G > A (21.1%), which shows the importance of this mutation to GBM development, since it constitutively activates the *TERT* gene, supporting the maintenance of genomic integrity through telomere elongation (Heidenreich *et al.* 2014; Walsh *et al.* 2015).

By analyzing an important dataset of gliomas, Ceccarelli *et al.* (2016) described distinct glioma subgroups based on methylation and gene expression status, and correlated them with survival, grade, and age at diagnosis. Based on DNA methylation analysis, the authors described six clusters: three clusters presented *IDH* mutations and were enriched for low-grade gliomas, whereas the clusters with wild-type *IDH* were enriched for GBMs. In fact, we found 77.8% (7/9) of diffuse and 100% (7/7) of anaplastic astrocytomas presenting *IDH1* mutation, whereas 92.9% (3/42) of GBMs were wild-type for *IDH1* mutation, corroborating this data. Independent of the tumor grade, we also found a dramatic increase in survival in patients presenting *IDH1* mutation (29.2 months), suggesting that this gene is an important biomarker, as the authors have previously found that *IDH* mutation was the main driver of the clusters (Ceccarelli *et al.* 2016).

Similarly, other comprehensive studies suggest that the combined analysis of the mutational status of *TERT*, *IDH*, and 1p/19q deletion had the ability to define the biological and clinical behavior of gliomas better than analysis based solely in histology (Foote *et al.* 2015; Eckel-Passow *et al.* 2015). When we performed this stratification in our samples, we found that the group presenting only *TERT* mutation had a dramatically reduced survival of 8.5 months *vs.* 29.2 months of only *IDH1*-mutated patients. This is consistent with recent data that showed an association of *TERT* mutation with poor survival, and that of *IDH1* mutation with better survival (Eckel-Passow *et al.* 2015; Foote *et al.* 2015). Generally, mean age at diagnosis in our groups was also consistent with the literature, with elderly patients presenting only *TERT* mutation (Eckel-Passow *et al.* 2015).

Besides the alterations in the genes extensively studied in GBMs, there may exist some less-studied regions/genes that could help in the understanding of GBM development and/or could be potential targets in GBM treatment. To identify these genes, we selected those present in regions frequently amplified or deleted in our GBMs and exploring their expression in Oncomine datasets. Hodgson *et al.* (2009) assessed the gene expression data of TCGA-derived GBMs and found overexpression of genes related to cellular assembly and organization and, among other genes, the authors found *CDK2* (located at 12q13), which was found amplified in four of our GBM samples. In fact, this gene interacts with others (found overexpressed in TCGA-derived samples), such as *AURKB*, *BIRC5*, *CCNB1*, *CCNB2*, *CDC2*, and *FOXM1*, and forms a transcriptional network important for G2/M progression and/or checkpoint activation (Hodgson *et al.* 2009). Still related to cell proliferation and differentiation, *DMRTA1* (chr9p21.3) and *DMRTA2* (chr1p32.3) were found deleted in 21 and two samples, respectively. These genes are highly expressed in the early developing telencephalon of rodent embryos (Kikkawa *et al.* 2013; Konno *et al.* 2012). Studies show that *DMRTA1* is a downstream gene of *PAX6*, a potent regulator of proliferation and differentiation of neural stem/progenitor cells. Once expressed, *DMRTA1* (together with *DMRTA3*) promotes neuronal differentiation via regulation of *NEUROG2* (Kikkawa *et al.* 2013). On the other hand, *DMRTA2* plays pivotal roles in the early development of the telencephalon via formation of the cortical hem, a source of Wnts, and by maintaining neural progenitors as a downstream target of the Wnt pathway (Konno *et al.* 2012).

The *MTAP* gene was codeleted with *CDKN2A* in 21 GBMs. The protein coded by *MTAP* cleaves MTA (generated during polyamine biosynthesis) in adenine, and it is converted to AMP and 5-metiltioribose-1-phosphate. Then, 5-metiltioribose-1-phosphate is converted to methionine (Bertino *et al.* 2011). Therefore, this protein is responsible for the recycling of adenine and methionine in the normal metabolism (Bertino *et al.* 2011). The role of MTAP in gliomas is poorly characterized. High frequency of *MTAP* deletion has been described in high-grade gliomas (Sasaki *et al.* 2003), namely in GBMs (Nakahara *et al.* 2004; Suzuki *et al.* 2004), in agreement with the present study, and also our recent report of MTAP protein expression in more than 85% of pilocytic astrocytomas (Becker *et al.* 2015b). Other studies have reported that homozygous deletion of *MTAP* is highly associated with loss of expression (Crespo *et al.* 2012), and that its expression is associated with lifetime- and progression-free survival in GBMs (Serao *et al.* 2011). Interestingly, we previously described that *MTAP* expression is possibly disrupted through intragenic breakpoints in pediatric high-grade gliomas (Carvalho *et al.* 2014).

Through bioinformatics approaches, we found that a family of several interferon (*IFN*) genes are coexpressed with *MTAP* and *CDKN2A*. In fact, these genes are located at the same cytoband and frequently deleted in GBM. Exogenous *IFN* has been used for biotherapy in several malignancies (Dillman 2011), since IFN treatment may induce apoptosis in tumor cells (Sgorbissa *et al.* 2011). Additionally, studies have evaluated the contribution of autocrine *IFN* production in the apoptotic response to *IFN*α in U87MG and T98G cells. They found that endogenous IFN production is responsible for sustaining high levels of TRAIL, and that loss of *IFN* genes confers an adaptive advantage to cancer cells, since they confer resistance to *IFN*α-induced apoptosis (Sgorbissa *et al.* 2011). In line with this, we found in the TCGA dataset that loss of expression of *IFNA13*, *IFNA21*, *IFNA6*, *IFNA8*, *IFNB1*, or *IFNW1* was correlated to poor survival, increasing the evidence for the importance of the tumor-stroma microenvironment interaction in gliomagenesis.

Despite the extensive molecular characterizations published worldwide, all patients are been treated using the same standard protocols and the outcome of high-grade gliomas remains poor (Malmstrom *et al.* 2012). To date, there are very few predictive biomarkers, with *MGMT* methylation status the only one to have been accepted by consensus and in clinical use (Hegi *et al.* 2005; Malmstrom *et al.* 2012). In 2014, the International Society of Neuropathology recommended, in the “ISN-Haarlem Consensus Guidelines,” the support of molecular analysis in the determination of tumor entities (Louis *et al.* 2014), showing the emerging importance of molecular analyses in diagnosis.

In conclusion, we performed, for the first time, an integrated characterization of chromosomal CNA, microsatellite instability, and *TERT*/*IDH1* mutational analysis in astrocytomas arising in the Brazilian population. Besides the expected similar pattern of alterations described worldwide, the combination of our findings with *in silico* analysis of the Oncomine and TCGA data has led to the identification of genes for further investigation in glioma, such as *CDK2*, *DMRTA1*, *MTAP*, and *IFN*. This study contributes to the molecular profiling of astrocytomas, and constitutes an important step towards future personalized medical approaches for the treatment of patients diagnosed with astrocytomas.

## Supplementary Material

Supplemental Material
