# Supplementary material for: Copy Number Profiling of Brazilian Astrocytomas
Source: G3 (Bethesda). 2016 Apr 25;6(7):1867–78. doi: 10.1534/g3.116.029884 (PMC4938641; doi:10.1534/g3.116.029884)
Supplement: Supplemental Material [file supp_6_7_1867__index.html]

Copy Number Profiling of Brazilian Astrocytomas — Supplemental Material 

# Copy Number Profiling of Brazilian Astrocytomas

## Supplemental Material for Bidinotto *et al.*, 2016

**Files in this Data Supplement:**

- Table S1 - Number of samples presenting mutation in *TERT* and *IDH1*. (.pdf, 147 KB)
