# Supplementary material for: Copy Number Profiling of Brazilian Astrocytomas
Source: G3 (Bethesda). 2016 Apr 25;6(7):1867–78. doi: 10.1534/g3.116.029884 (PMC4938641; doi:10.1534/g3.116.029884)
Supplement: Supplemental Material [file supp_g3.116.029884_TableS1.pdf]

Table S1 – Number of samples presenting mutation in *TERT* and *IDH1*.

Tumor type	*TERT*		*IDH1*	
	-124C>T	-146C>T	Arg132His	Arg132Cis
Pilocytic astrocytoma	0	1 (14.3%)	0	0
Diffuse astrocytoma	1 (11.1%)	0	6 (66.7%)	1 (11.1%)
Anaplastic astrocytoma	0	0	5 (71.4%)	2 (28.6%)
Glioblastoma	21 (50%)	8 (19.1%)	3 (7.7%)	0

Values expressed in number or mutated cases (percentage) in each tumor type.
